# MSC Therapies for COVID-19: Importance of Patient Coagulopathy, Thromboprophylaxis, Cell Product Quality and Mode of Delivery for Treatment Safety and Efficacy

**DOI:** 10.3389/fimmu.2020.01091

**Published:** 2020-05-19

**Authors:** Guido Moll, Norman Drzeniek, Julian Kamhieh-Milz, Sven Geissler, Hans-Dieter Volk, Petra Reinke

**Affiliations:** ^1^BIH Center for Regenerative Therapies (BCRT), Charité Universitätsmedizin Berlin, Berlin, Germany; ^2^Berlin-Brandenburg School for Regenerative Therapies (BSRT), Charité Universitätsmedizin Berlin, Berlin, Germany; ^3^Institute of Medical Immunology, Charité Universitätsmedizin Berlin, Berlin, Germany; ^4^Department of Transfusion Medicine, Charité Universitätsmedizin Berlin, Berlin, Germany; ^5^Julius Wolff Institute (JWI), Charité Universitätsmedizin Berlin, Berlin, Germany; ^6^Department of Nephrology and Internal Intensive Care Medicine, Charité Universitätsmedizin Berlin, Berlin, Germany; ^7^Berlin Center for Advanced Therapies (BECAT), All Charité Universitätsmedizin Berlin, Corporate Member of Freie Universität Berlin, Humboldt-of Universität zu Berlin, Berlin Institute of Health (BIH), Berlin, Germany

**Keywords:** mesenchymal stromal cells (MSC), severe acute respiratory distress syndrome coronavirus-2 (SARS-CoV-2), coronavirus-induced disease 2019 (COVID19), intensive care unit (ICU), intravascular and intravenous infusion, hemocompatibility testing, tissue factor (TF/CD142), coagulation/clotting/thrombosis

## Abstract

Numerous clinical trials of mesenchymal stromal/stem cells (MSCs) as a new treatment for coronavirus-induced disease (COVID-19) have been registered recently, most of them based on intravenous (IV) infusion. There is no approved effective therapy for COVID-19, but MSC therapies have shown first promise in the treatment of acute respiratory distress syndrome (ARDS) pneumonia, inflammation, and sepsis, which are among the leading causes of mortality in COVID-19 patients. Many of the critically ill COVID-19 patients are in a hypercoagulable procoagulant state and at high risk for disseminated intravascular coagulation, thromboembolism, and thrombotic multi-organ failure, another cause of high fatality. It is not yet clear whether IV infusion is a safe and effective route of MSC delivery in COVID-19, since MSC-based products express variable levels of highly procoagulant tissue factor (TF/CD142), compromising the cells' hemocompatibility and safety profile. Of concern, IV infusions of poorly characterized MSC products with unchecked (high) TF/CD142 expression could trigger blood clotting in COVID-19 and other vulnerable patient populations and further promote the risk for thromboembolism. In contrast, well-characterized products with robust manufacturing procedures and optimized modes of clinical delivery hold great promise for ameliorating COVID-19 by exerting their beneficial immunomodulatory effects, inducing tissue repair and organ protection. While the need for MSC therapy in COVID-19 is apparent, integrating both innate and adaptive immune compatibility testing into the current guidelines for cell, tissue, and organ transplantation is critical for safe and effective therapies. It is paramount to only use well-characterized, safe MSCs even in the most urgent and experimental treatments. We here propose three steps to mitigate the risk for these vulnerable patients: (1) updated clinical guidelines for cell and tissue transplantation, (2) updated minimal criteria for characterization of cellular therapeutics, and (3) updated cell therapy routines reflecting specific patient needs.

## The Promise Of Msc Therapies As Treatment For Covid-19

**Figure 1:**
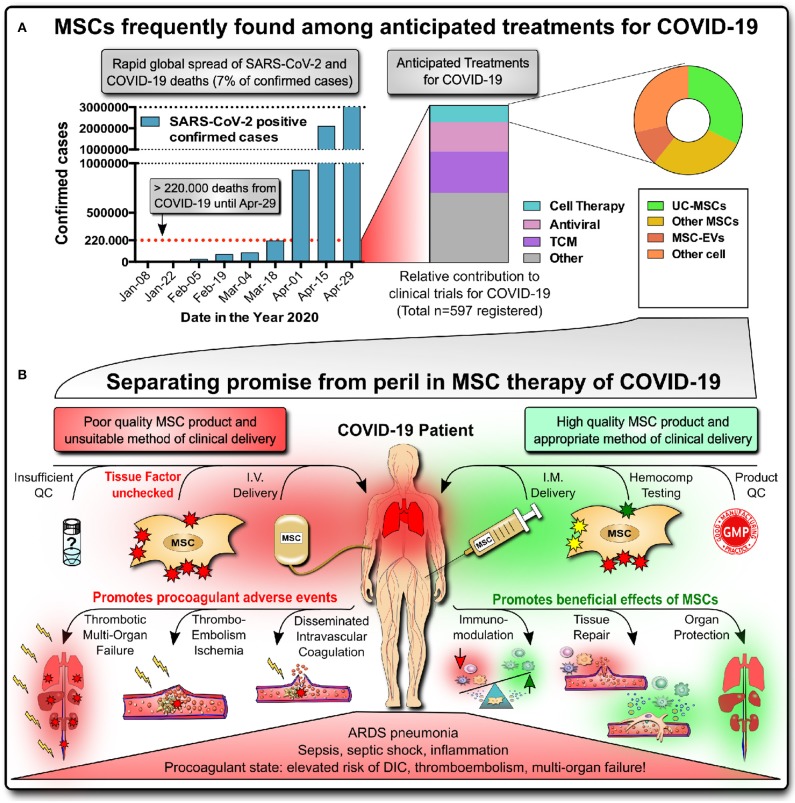
Promise of MSC therapies for COVID-19. **(A)** Rapid global spread of severe acute respiratory syndrome-coronavirus-2 (SARS-CoV-2) infection, reaching >3 million confirmed infected cases and >220,000 deaths (7% of total) by coronavirus-induced disease 2019 (COVID-19) by April 29, 2020, according to John Hopkins University of Medicine (https://coronavirus.jhu.edu/map.html). Newly registered clinical studies for COVID-19 frequently show MSCs, particularly umbilical cord (UC)-derived MSC products, to be among the anticipated treatments for critically ill patients (the list of 597 studies with status as of April 14, 2020, was compiled by Cell-Trials-Data (30); TCM, traditional Chinese medicines; EC, extracellular vesicle). **(B)** Separating promise from peril in MSC therapy of COVID-19. Critically ill COVID-19 patients suffering from acute respiratory distress (ARDS) pneumonia, inflammation/sepsis, and a systemic procoagulant state are at elevated risk for disseminated intravascular coagulation (DIC), venous thromboembolism and thrombotic multi-organ-failure. While high-quality MSC products applied via intramuscular (IM) injection hold promise to cure COVID-19 by exerting beneficial immunomodulatory effects, tissue repair and organ protection, the worst-case scenario of intravascular (IV) infusion of high doses of poorly characterized MSC products with unchecked/high tissue factor TF/CD142 expression can potentially promote adverse events and lead to potentially lethal embolism and thrombotic multi-organ failure.

Coronavirus-induced disease 2019 (COVID-19) has brought many intensive care units (ICUs) in hotspots of severe acute respiratory syndrome-coronavirus-2 (SARS-CoV-2) infection throughout China, Europe, and America to the brink of collapse in the past months, and the virus continues to spread rapidly throughout the globe (Figure 1A, left panel) (1–3). To date, no approved effective therapy is available that can halt the progression of COVID-19 and can address the critical cases with high fatality, driving public fear in the “Corona Crisis.” Thus, any treatment that could reduce case fatality by alleviating severe COVID-19 and speed up the recovery of critically ill patients is in great demand, with advanced mesenchymal stromal cell (MSC) therapeutics holding promise to fulfill this need (4–8).

In the majority of patients, SARS-CoV-2 infections range from being asymptomatic to seasonal flu-like symptoms, while ~14% of cases presented with severe outcomes and ~6 and ~3% with critical and fatal outcomes, respectively (9, 10). The severe cases require ICU care due to lung and multi-organ failure, being associated with tissue damage and a virus-induced cytokine storm with a distinct pattern (11–14). Depending on patient sex/age, comorbidities, and available ICU capacity, mortality in the critical ventilated patient population with respiratory failure has been reported to be as high as 50%—with sepsis or septic shock a leading cause of death (14–16). Another major concern is the abnormal coagulation profile seen in many critically ill ICU patients in potential need of MSC therapy (15–26).

Due to their multifactorial mode-of-action (MoA), MSC therapeutics are perceived to be ideal candidates for tackling the broad spectrum of COVID-19 symptoms and are now in great demand, counting >20 active clinical MSC trials (Figure 1A, right) (27–31). MSC therapies have shown promising results in the treatment of acute respiratory distress syndrome (ARDS) and sepsis, but efficacy data are still scarce in humans (Table S1) (32–35). Major manufacturers of advanced MSC-like therapeutics have registered trials for COVID-19 (e.g. Athersys, Mesoblast, and Pluristem) (36, 37). However, it is also evident that many early-stage operations are trying to market poorly characterized unregulated MSC treatments, thus being sanctioned by organizations such as ISCT, ISSCR, and EMA (27–29, 38–40).

Various MSC therapies, from small investigator-driven studies to advanced industrial-scale manufacturers with global marketing capacity, have been explored in preclinical animal models, human case studies and early phase trials for ARDS, acute lung injury (ALI), and sepsis (Table S1, part 1) (32–35). Some prominent examples include two interesting case studies from Sweden (32, 41, 42), the START phase 1 and 2 trials (35, 43–45), and the SEPCELL phase 1 and 2 trials (46), along with many newly registered trials for COVID-19 (Table S1, part 2) (27–31). Noteworthy, so far only few of the cell therapy studies for ARDS and sepsis have been shown to meet their primary endpoints in randomized studies (29, 35).

Although first case reports on MSCs for COVID-19 gathered during the early outbreak phase in China provide valuable hints that the treatment may be somewhat safe and efficacious, experts agree that proper clinical investigations are now essential (27–29, 47). Conclusions from these first studies are limited due to the small number of included patients (typically no more than 10) and the lack of adequate control groups (48–50). Proper clinical trial design and adherence to quality measures, such as documentation of included patients, inclusion/exclusion criteria, stratification of treatment arms, primary and secondary readouts, and timing and dosing regimens of treatments and comedication, are urgently needed (47, 51).

Although early results might appear promising, one should be reminded of both the previous failures of advanced clinical studies with MSCs and the low level of approved MSC products (5, 8, 52–54). Multiple problems were identified, such as failures in up-scaling the product manufacturing to large-scale supply and a loss in translation to effective clinical application (e.g., degree of cell expansion from limited starting material, cell viability issues post-thawing, and suboptimal route of delivery) (7, 35, 55–57), which may explain study failures (5, 8). If some of the advanced phase II/III clinical studies produce more solid evidence supporting product approval in the months to come (as discussed below), another key issue for sustainable marketing will be the technological readiness level of the products and their manufacturers (52, 53). The dynamics of the pandemic virus spread and the rising number of global deaths make it clear that major manufacturing and sound logistic capacity are needed to supply sufficient doses of high-quality cell product in a reproducible and timely manner.

## Hypercoaguability In Covid-19 Patients With Poor Prognosis Mandates Great Caution With Iv Delivery Of Msc Therapeutics

The most frequently anticipated form of cell product delivery in ARDS and COVID-19 is the intravenous (IV) infusion of MSCs with the primary aim of targeting the lungs (6–8). It is not yet clear if this is a safe and effective route of cell delivery in COVID-19, considering that MSC products express variable levels of highly procoagulant tissue factor (TF/CD142) (58), compromising the cells' hemocompatibility and safety profile (Figure 1B) (6–8, 59–61). Numerous clinical reports indicate (15–26) that many of the critically ill COVID-19 patients with poor prognosis are in a systemic procoagulant state at high risk of disseminated intravascular coagulation (DIC), thromboembolism, and thrombotic multi-organ failure, one of the leading causes of death in these patients. This would make IV applications of MSCs a contraindication in COVID-19 due to the potential harm to these patients (6–8, 59–61).

A first study reported in February 2020 by Dr. Ning Tang et al. from Wuhan, China (17, 18) found that 71.4% of non-survivors compared to 0.6% of survivors in a cohort of 183 consecutively included COVID-19 patients met the ISTH diagnostic criteria for overt DIC (≥5 points) (17, 62). This included significantly elevated levels of D-dimer (>1 μg/mL) (22) and fibrin-degradation product and longer prothrombin time (PT) and activated partial thromboplastin time (APTT). The median time from admission to DIC was 4 days, and it was evident that abnormal coagulation parameters (e.g., elevated D-dimer) may act as potential predictors of a poor prognosis.

In a larger cohort reported in April 2020 by Dr. Tao Wang on behalf of the National Clinical Research Center for Respiratory Disease and the National Health Commission of the People's Republic of China (19), ~40% of COVID-19 patients (407 of the 1,026 included cases) were at high risk of thromboembolism. It was estimated that 11% of these high-risk patients develop venous thromboembolism without appropriate prophylaxis (63), but only a few (7%) of the patients were given blood thinners (mainly heparin) during hospitalization (19). In total, 11% (44 of 407) of patients at high risk for thromboembolism were also at high risk for bleeding, which may explain the hesitation to use anticoagulants. It was recommended that for these patients, the dose and duration of anticoagulants should be carefully adjusted.

The risk for thromboembolism is further substantiated by a case study of three COVID-19 patients managed by a team from Peking Union Medical College Hospital at Tongji Hospital in Wuhan, China (20). The first patient presented with ischemia in the lower limbs and in two digits of the left hand. Computed tomographic imaging of the brain showed bilateral cerebral infarcts in multiple vascular territories. Laboratory analysis documented leukocytosis, thrombocytopenia, elevated PT and APTT, and elevated levels of fibrinogen and D-dimer. Serological testing revealed the presence of antiphospholipid antibodies, which can arise transiently in patients with critical illnesses and infections. Two other patients with similar findings were seen in the ICU for COVID-19 patients at Tongji Hospital.

These early reports from China have been confirmed by a Dutch multi-center study incorporating 184 ICU patients who received standard doses of thromboprophylaxis at hospital admission (21, 22). Klok et al. still documented thrombotic complications in 31% of patients and emphasized the strict need for thromboprophylaxis in all COVID-19 patients admitted to the ICU (21). Others also suggested targeting both the prothrombotic state and complement-activation-induced microvascular injury in the pathogenesis of severe COVID-19 infection (23, 64). Several newly initiated studies are now investigating the optimal dosing of thromboprophylaxis for the prevention of clot formation, and, alternatively, also treatments that can dissolve already existing clots with agents such as tissue plasminogen activator and antithrombotic therapies typically reserved for the treatment of strokes and heart attacks (25, 26, 65).

In conclusion, preliminary data on COVID-19 indicate a substantial risk that infusions of TF/CD142-expressing MSC products could aggravate the pro-thrombotic state of COVID-19 (and other categories of patients in a hypercoagulable state) and increase the risk of associated complications such as DIC, thrombosis, and thrombotic multi-organ failure (7, 19, 20, 59). We here wish to raise awareness to this safety issue to raise awareness to this safety issue, provide scientific context, and propose three steps for improved product characterization, optimized product delivery, and comprehensive integration of innate hemocompatibility testing for IV-applied cellular therapies into clinical practice, as outlined in the following paragraphs.

## Need For Hemocompatibility Testing Of Iv Applied Cellular Therpeutics And Alternative Routes Msc Delivery In Clinical Trials

**Figure 2:**
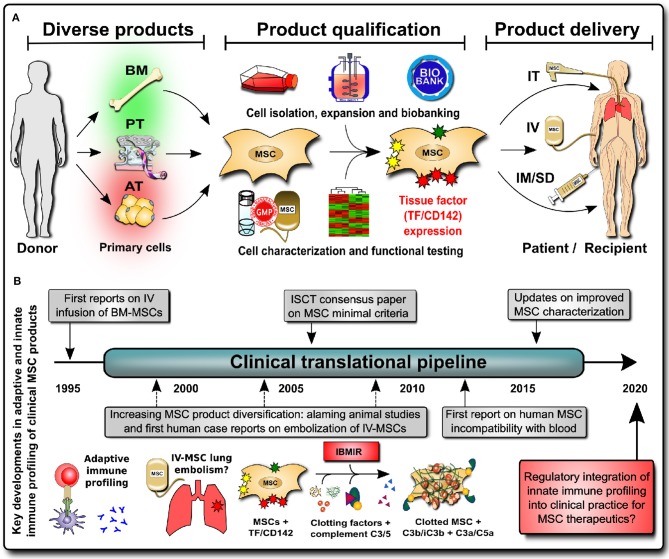
Integrating innate immune profiling of cell therapies into clinical practice. **(A)** MSC products have greatly diversified (e.g., the tissue source that they are derived from, with bone marrow (BM), perinatal tissue (PT), and adipose tissue (AT) being the most frequent sources), and product qualification has shown large differences in expression of the highly procoagulant tissue factor TF/CD142 between products (BM lowest, PT intermediate, and AT highest), which impacts on the cell hemocompatibility and preferred mode of clinical product delivery to patients [e.g., intravenous (IV) infusion vs. intramuscular or subdermal (IM/SD) injection or intratracheal (IT) direct pulmonary delivery with bronchoalveolar lavage (BAL)]. **(B)** Historical timeline of integrating innate immune profiling of cellular therapies into clinical practice to mitigate the risk for potentially lethal thromboembolism due to triggering of the instant blood-mediated inflammatory reaction (IBMIR) upon intravascular/intravenous (IV) infusion; C3/C5, complement factors 3 and 5; C3a/C5a, complement activation fragmentss 3a and 5a.

It is apparent that there is an urgent clinical need for new guidelines on hemocompatibility testing for IV-delivered cellular therapeutics for two major reasons (Figure 2) (7, 8, 59–61) (1) the varying risk profiles of patients considered for treatment with IV-MSC therapeutics, and (2) the difficult-to-predict risk profiles of the different clinically available MSC products. Differences may also exist in the quality of MSC therapeutics and their mode of delivery when comparing products from major well-established manufacturing centers that have many years of experience with poorly documented unregulated small-scale operations that produce products with unknown properties (e.g., as a result of batch-to-batch inconsistency) due to poor standard operating procedures and a distinct lack of clinical routines.

First of all, the risk profile of patients differs greatly due to the large diversity of indications and concomitant hemostatic profiles, particularly in patients with hypercoagulability (66). The prior example of COVID-19 made it clear that particularly critically ill patients with a poor prognosis in potential need of MSC-therapy are in a highly activated hypercoagulable state and thus at risk of dying from DIC, embolism, and thrombotic multi-organ-failure. The same applies to other pre-activated patient indications, such as severe trauma and sepsis, and in patients with considerable comorbidities, such as advanced diabetes and renal failure. Indeed, several reports already documented cases of DIC and thromboembolism occurring after the infusion of TF/CD142-expressing MSC products, particularly in preactivated patients (7).

The second issue is the varying risk profiles of different MSC products, e.g., depending on the degree of TF/CD142 expression and the anticipated route of clinical delivery. MSC treatments greatly diversified prior to the start of the SARS-CoV-2 pandemic (7), and available treatments differ greatly in their hemocompatibility (Figure 2A, left panel). The initial safety profiles for MSC infusions were established with bone marrow (BM)-derived MSCs, which have low TF/CD142 expression, but nowadays, we have approximately equal shares of three major sources: BM-, adipose tissue (AT)-, and perinatal tissue (PT)-derived MSC products. All three differ greatly in their expression of highly procoagulant TF/CD142, thus affecting their safety and efficacy profiles and the preferential route of clinical delivery (Figure 2A, center) (7).

Appreciating the complexities surrounding both patients and the background of MSC-products highlights the danger of utilizing poorly characterized experimental products with unchecked/high TF/CD142 expression. This could prove particularly problematic for those patients that suffer from a dysregulation of the hemostatic system (66). George et al. reported that blood clotting in trauma patients in a state of hypercoagulability was accelerated by commonly used IV-infused cellular therapeutics in relation to the degree of TF/CD142 expression in the product (8, 60, 61, 66). To illustrate that this is not just a hypothetical risk, peripheral microthrombosis, embolism, and even potential cases of death have already been documented in patients that received infusions of highly TF/CD142-expressing MSCs (67–69), and it is expected that similar cases may arise as a result of MSC infusion in COVID-19.

Alternative routes of cell administration such as intramuscular (IM) injection are increasingly explored as alternatives to IV injection (Figure 2A, right) because of longer *in vivo* survival of the cells, improved functionality, and a lack of hemocompatibility issues (8, 57, 70–73). Galipeau et al. found that potency is dependent on the route of cell delivery, cell viability, and immune match (57) and that the mode of delivery impacts strongly on MSCs' therapeutic activity (73). IM delivery potentiates the dwell time of MSCs due to the favorable *in vivo* milieu (8, 70, 72). The highly vascularized muscle tissue serves as a physiological environment able to supply the therapeutic cells with oxygen and nutrients and to safeguard their prolonged survival while also supporting their prolonged secretion of beneficial paracrine mediators.

The integrated understanding of product properties, patient background, and optimal cell delivery is crucial for the safe and effective use of MSCs and other products (6–8). The preferential use of well-characterized products from robust manufacturing sources with optimized modes of delivery [e.g., careful consideration of intravascular (IV) vs. intramuscular (IM) vs. intratracheal (IT) modes of delivery depending on product properties] and appropriate adjunct patient treatment protocols (e.g., suitable anticoagulation and other comedication) may greatly mitigate any risk for patients and allow MSCs to live up to their full potential. These high-quality cell products may become valuable therapeutics (6), in contrast to poorly characterized cell products with high batch-to-batch heterogeneity and unsuitable protocols for clinical application, which may pose a risk to patients.

## Weighing Risk and Benefit of Intravenous Vs. Intramuscular Cell Application Considering MSC Treatment Safety, Efficacy, and Proposed Mechanism of Action

Considering risk-benefit approximation, the priority in early-phase trials is clearly safety, with a reasonable but careful dose-escalation. Importantly, higher dosing is usually assumed to be beneficial in clinical trials due to a perceived increase in active agent/treatment potency. However, the detrimental worst-case combination of infusing highly TF/CD142-expressing MSCs at high doses into hypercoagulable patients should clearly be avoided, being a potential contraindication in COVID19 and thus clearly a dose-limiting factor. In contrast, MSCs with low or absent TF/CD142 may be suitable for IV delivery in hypercoagulable patients with appropriate treatment protocols (e.g., suitable anticoagulation), making the MSC tissue source and the intrinsic cellular potency one of the decisive factors (7). Advanced trials need to carefully weigh the risk to patients resulting from adverse events or treatment failure (e.g., lack of efficacy) vs. short- and long-term benefits for the patient (35), requiring sufficient product potency/efficacy and appropriate measures thereof in patients (Table S1).

A collection of higher study endpoints extracted from Table S1 includes parameters such as: (1) *respiratory function* (e.g., oxygen index 3 days after MSC infusion or measured by chest computerized tomography at days 2 and 14), and (2) *mortality/survival* [e.g., at days 14 and 28 (death by any cause) and ICU/hospital stay at day 28 (total duration), ideally with long-term 1-year follow-up], (3) *lung mechanics and ventilator weaning* (e.g., arterial oxygen saturation, tidal volume, minute ventilation, ratio PaO_2_/FiO_2_, failure of ventilator weaning, weaning time, and ventilation time), (4) *hemodynamic parameters* (e.g., systolic, diastolic, and mean arterial blood pressure), (5) *inflammation and infection* (may differ for viral and bacterial, e.g., plasma cytokines IL-1, IL-6, IL-8, IL-10, or only IL-6/8 with early monitoring 6 h post-MSC and at days 1, 2, and 3, then also lactate, DIC score, SOFA score, C-reactive protein, and procalcitonin), and (6) *lowering lung fibrosis* (important for recovering lung-capacity in “cured” patients and hence enabling future ability to return to job and reducing health care costs for survivors). We here wish to give a short outline of the preliminary results of some representative clinical studies from major well-established and regulated manufacturers, as recently compiled by CellTrials.Org (29).

In the newly registered advanced COVID-19 trials of Athersys (MAPC-/BM-MSC-based MultiStem® product) and Mesoblasts (BM-MSC-based), the cell product is delivered IV. These cell products have low TF/CD142 expression (6–8, 74), which may be tolerated with appropriate adjunct infusion protocols and well-defined patient inclusion/exclusion criteria (e.g., excluding pre-history of patient coagulopathy). Both Athersys and Mesoblast reported preliminary safety and efficacy in preceding studies and have now advanced to phase II/III studies with prior approval by the corresponding regulatory authorities such as the FDA (29).

AT-derived MSCs are among the highly TF/CD142-expressing cell products, and careful dose-escalation by TiGenix/Takeda in their SPECELL study (AT-MSCs Cx611 product) has shown a significant increase in the coagulation activation markers thrombin-antithrombin-complex (TAT) and D-dimer for infusion of 4 million cells/kg vs. controls in healthy volunteers with normal coagulation parameters (75). Accordingly, the dose-limiting toxicity should be lower in hypercoagulable COVID-19 patients, potentially limiting the cell dose to <4 million cells/kg.

The TF/CD142 load of a given MSC product may be of less or no importance for IM and IT delivery due to the delivery of the cells into the extravascular space (avoiding blood contact), therefore allowing for higher cell doses than IV infusions without dose-limiting toxicity. Pluristem typically employs IM injection of high cell doses of placenta-derived MSC-like cells (typically up to 300 million cells/patient are used, but also higher doses can easily be applied without apparent safety concerns), and preliminary data from eight patients treated in Israel and the United States have shown good safety and efficacy, thus also proceeding to phase II/III studies.

In conclusion, while the primary risk outlined earlier in this perspective is clear perspective is clear, the potential benefit is more difficult to assess/define in ARDS and COVID-19 due to the current lack of efficacy data and the general need for a more clearly defined MoA (33). Considering potency and efficacy, it has been speculated that close proximity of the cells to the major sites of pathological damage (such as the lungs) may be of advantage, though this is yet to be proven due to the complex MoA. A clear advantage of IM or IT over IV delivery lies in the higher effective cell dose that can be applied to patients, thus increasing the amount of active agent and along potentially also the treatment potency and efficacy. Either way, decision-making is reliant on quantifying the TF/CD142 expression of MSCs and testing their hemocompatibility before clinical use.

## Integrating Hemocompatibility Testing of Cellular Therapeutics Into Clinical Practice

More than 60 years ago, a great collaborative effort by Donall Thomas and his contemporary colleagues laid the foundation for modern transplant medicine through understanding the adaptive immune mechanisms underlying transplant incompatibility between humans (76). Once the mechanisms of recipient-donor human leukocyte antigen (HLA) incompatibility and matching were understood, transplantation of cell/tissue/organ grafts became feasible. Nowadays, well over 100,000 (allo)-transplantation procedures are performed annually worldwide, and they are regulated by, amongst others, the World Health Organization (WHO) and their Guiding Principles on Cell, Tissue, and Organ Transplantation (77). Considerations regarding adaptive immune compatibility testing in MSC characterization for clinical use were, as such, integrated into clinical practice at an early stage (Figure 2B, left) (78).

More recently, the importance of innate immunity has been recognized in transplantation, e.g., in ischemia-reperfusion injury (IRI)-induced graft failure (79). In addition to cellular/humoral alloimmune-responses, innate incompatibility reactions induce/promote graft failure through rapid triggering of innate immune cascades (e.g., complement/coagulation activation and concomitant thrombotic reactions (Figure 2B, center) (7, 80–83). This detrimental cascade has been termed “instant blood-mediated inflammatory reaction” (IBMIR), and the expression of tissue factor (TF/CD142) has been identified as a key trigger of IBMIR, e.g., in IV transplantation of islet of Langerhans cell clusters and therapeutic MSCs (7, 80, 83, 84).

MSCs are one of the most widely used IV cell therapies of non-hematopoietic origin, and according to the ISCT minimal criteria (85), they are characterized by three major features: (1) plastic-adherent fibroblast-like morphology, (2) differentiation into multiple “mesenchymal” tissue lineages, and (3) presence or absence of specific cell surface markers. Recent efforts demonstrate that the minimal criteria can be adjusted according to specific clinical needs, such as allowing for the integration of MSC immune functional assays as a potency release criterion for advanced-phase clinical trials (86). We thus propose to update the panel of cell surface markers used to better characterize IV MSC therapies through the inclusion of a minimal set of markers indicative of hemocompatibility, and this would mainly encompass the expression of the highly procoagulant TF/CD142 (Figure 2B, right). In addition, standardized *in vitro* and *in vivo* hemocompatibility testing should be conducted for all new IV-applied MSC(-like) products and other cellular therapeutics prior to application in clinical trials. Cellular therapeutics differ greatly in TF/CD142 expression (7, 8, 59–61), but their hemocompatibility is not yet tested even when applied to patients via IV delivery (85). Thus, the risk of (lethal) thrombotic complications is most apparent if clinicians are not fully aware of this problem, and it is imperative that they are aware of said risks to enable the use of appropriate countermeasures (e.g. anticoagulation, if appropriate in a given patient indication) or the choice of more appropriate treatment options and application routes (e.g., IM instead IV injection) (7). We propose three critical steps to guarantee safe and effective cellular therapeutics in the future.

## Recommendations

1. Updated Clinical Guidelines for Cell and Tissue Transplantation: Integration of essential considerations on hemocompatibility testing into the current clinical guidelines for cell and tissue transplantation in addition to well-established recommendations considering all aspects of allo-immunogenicity and other testing and in line with standards for hemocompatibility testing of medical devices in contact with blood (e.g., WHO recommendations on human cell and tissue transplantation and ISO10993-1/4 guidelines for medical devices) (77, 82, 87).
2. Updated Minimal Criteria for Characterization of Cellular Therapeutics: According to the intended mode of therapeutic cell delivery, hemocompatibility testing should be mandatory for all cellular therapeutics applied via intravascular delivery, particularly for non-hematopoietic cells not typically found in contact with blood (e.g., incorporation of TF/CD142 expression monitoring for therapeutic MSCs into the WHO recommendations and/or the International Society for Cellular Therapy (ISCT) minimal criteria) (7, 8, 77, 82, 88).
3. Updated Cell Therapy Routines Reflecting the Specific Patient Needs: The clinical cell product properties and mode of cell-delivery should anticipate the specific patient needs under consideration of the target indication to be treated (e.g., anticipation of anticoagulation protocols/bleeding risk and IM application as an alternative to IV infusion, shown to result in longer cell survival *in vivo*, associated with prolonged secretory activities, and a lack of coagulation issues, which may be important in the treatment of COVID-19) (7, 8, 60, 73).

## Conclusion

MSC products are rapidly emerging as promising treatment candidates for COVID-19 in the ongoing SARS-CoV-2 pandemic. They are being currently extensively explored both by leading manufactures and in many small investigator-initiated trials. Although cellular therapeutics are already widely employed in both preclinical and clinical settings, they can differ greatly in their hemocompatibility aspects, and they have been only poorly characterized in this regard so far. In order to minimize the evident risk of (lethal) adverse thrombotic reactions upon infusion of high doses of poorly characterized unregulated cell products, we have here proposed three decisive steps for integrating innate immune hemocompatibility testing into the standard characterization and clinical routines or IV applied cell therapies, and we also encourage the considerations of alternative non-intravascular application regimes, which may prove to be safer and more efficient alternatives in the long-run.

## Data Availability Statement

All datasets presented in this study are included in the article/Supplementary Materials.

## Author Contributions

All authors listed have made a substantial, direct and intellectual contribution to the work, and approved it for publication.

## Conflict of Interest

The authors declare that the research was conducted in the absence of any commercial or financial relationships that could be construed as a potential conflict of interest.
