# Supplementary material for: MSC Therapies for COVID-19: Importance of Patient Coagulopathy, Thromboprophylaxis, Cell Product Quality and Mode of Delivery for Treatment Safety and Efficacy
Source: Front Immunol. 2020 May 19;11:1091. doi: 10.3389/fimmu.2020.01091 (PMC7249852; doi:10.3389/fimmu.2020.01091)
Supplement: Supplementary file 1 [file Data_Sheet_1.PDF]

Table S1 Part 1: Mainly completed well-documented MSC studies for ARDS pneumonia and Sepsis/Septic-Shock.

Cell source	NCT # & Target Indication	Location	Short description of the study	Primary outcome Measures	Study status and expected date and references
**Allogeneic PT-MSC/DSCs**<br>(Perinatal tissue (PT)-derived mesenchymal stromal cells, specifically Placenta-derived decidual stromal cells)	**Case Study**<br>**Indication ARDS**<br>(Institutional Ethical Review Board of Karolinska Institutet: 2009/4-18-31/4, 2010/452-31/4)	Stockholm, Sweden	Compassionate basis<br>N=1 man with chronic myeloid leukemia developed acute lung injury (ALI) after allogeneic hematopoietic cell transplantation (HSCT) and sepsis. He became hypoxic and chest radiography suggested acute respiratory distress syndrome (ARDS).<br>**Case report published in 2014.**	- Oxygen saturation improvement<br>- Chest radiography improvement<br>- Systemic levels of proinflammatory cytokines/chemokines	**2014: First case report by Ringdén et al. on the use of placenta-derived MSC/DSC in treatment of ARDS by the Karolinska study group (*1, 2*).**<br>Results - After infusion of 1×106 DSCs/kg on day +11 after transplantation, oxygen saturation instantly increased from 92% to 98% and stabilized. The requirement for oxygen decreased and was discontinued on day +16. Chest radiography improved and normalized. Elevated systemic levels of proinflammatory cytokines/chemokines G-CSF, IL-6, IL-8, MCP-1, and TNF-α decreased. The patient was discharged on day +22 with normal chest radiography. He is alive and well nine months after transplant.
**Allogeneic BM-MSCs**<br>(Bone marrow (BM)-derived mesenchymal stromal cells)	**Case Study**<br>**Indication ARDS**	Stockholm and Uppsala, Sweden	Compassionate use<br>N=2 patients with severe refractory acute ARDS already deteriorated to be dependent on support by extracorporeal membrane-oxygenation (ECMO);<br>Both patients received $2x10^6$ cells per kilogram.<br>**Case report published in 2015.**	- Improved with resolution of respiratory,<br>- Improved hemodynamic,<br>- Improved multi-organ failure<br>- Decrease in markers of inflammation<br>- Decrease of proinflammatory cytokines, microRNAs, and chemokines	**2015: Second case report from the same study group on the use of BM-derived MSCs in treatment of ARDS; Conclusion: the placenta DSCs appear to be more promising (*1, 3*).**<br>Results - Both patients subsequently improved with resolution of respiratory, hemodynamic, and multi-organ failure. In parallel, a decrease was seen in multiple pulmonary and systemic markers of inflammation, including epithelial apoptosis, alveolar-capillary fluid leakage, and proinflammatory cytokines, microRNAs, and chemokines

**Allogeneic BM-MSCs**<br>(Bone marrow (BM)-derived mesenchymal stromal cells,	**NCT01775774**<br>**Indication ARDS**<br>Stem Cells for ARDS Treatment (START) phase 1	San Francisco and Boston, USA	Prospective, multicenter, open-label, dose escalation, phase 1 clinical trial, Patients enrolled in the ICU of Stanford University of California, USA, and Massachusetts General Hospital in Boston, USA<br>**Dose escalation 3x3 design:** a) low dose (1mio/kg), b) medium dose (5mio/kg) and c) high dose (10mio/kg)<br>**Patient recruitment between July 8th 2013 and January 13th 2014**.	- Patients were included if they had moderate-to-severe ARDS as defined by the acute onset of the need for positive pressure ventilation by endotracheal or tracheal tube.<br>- Primary outcomes included incidence of pre-specified infusion-associated events and serious adverse events	**2014: Phase-1 study completed in July 2014 and published in Lancet Respiratory Medicine online in 17th December 2014, preceded by two study primers / review articles (*4-7*).**<br>Results – No prespecified infusion-associated events or treatment-related adverse events in any of the 9 patients. Serious adverse events: in three patients during weeks after infusion: one died on study day 9, one died on study day 31, and one had multiple age-indeterminate embolic infarcts in spleen, kidneys, and brain, thought to have occurred before MSC infusion.<br>Interpretation – a single intravenous infusion of allogeneic BM-MSCs was well tolerated in nine patients with moderate-to-severe ARDS. Decision to proceed to phase 2 testing for ARDS with primary focus on safety and secondary outcomes including respiratory, systemic and biological endpoints
**Allogeneic BM-MSCs**<br>(Bone marrow (BM)-derived mesenchymal stromal cells)	**NCT02097641**<br>**Indication ARDS**<br>Stem Cells for ARDS Treatment (START) phase 2a	Multicenter, USA	Prospective, double-blind, multicenter, randomized trial of intravenous MSCs compared to placebo. Recruitment in 5 university medical centers in the USA;<br>**Dosing:** patients were randomized 2:1 to receive either 10mio/kg MSCs or placebo;<br>**Patient recruitment between 24th of March 2014 and 9th of Feb 2017.**	- Recruitment of ventilated patients with moderate-to-severe ARDS<br>- Exclusion of patients with trauma or moderate-to-severe liver disease and those who had cancer treatment in past 2years.<br>- 1038 patients screened of whom 60 were eligible and received treatment.<br>- Primary endpoint was safety, secondary endpoint included biomarkers in plasma.	**2019: Phase-2a study completed in February 2017 and published in Lancet Respiratory Medicine online 16th of November 2017, accompanied by commentaries (*8-10*).**<br>Results- No patient experienced any of the predefined MSC-related hemodynamic or respiratory adverse events. One patient in the MSC group died within 24h of MSC infusion, judged to be unrelated to MSC infusion; 28-day mortality did not differ between groups.<br>Interpretation – A single dose of intravenous BM-MSCs was safe in patients with moderate-to-severe ARDS. Larger trials are needed to assess efficacy. MSC viability ranging from 36-85% must be improved.

**Autologous BM-MSCs**<br><br>(Bone marrow (BM)-derived mesenchymal stromal cells)	**NCT02112500**<br><br>**Indication ARDS**<br><br>(STELLAR pilot to assess the efficacy and safety of MSCs in acute severe respiratory failure)	Sang Bum Hong, Asan Medical Center	Phase II, open label, single group assignment of intravenously infused BM-MSCs from patients.<br><br>**Dosing schedule not clearly indicated.**<br><br>**Estimated recruitment of 10 participants between February 2014 and December 2016.**	- Primary outcome oxygen index at 3 days after MSC infusion with oxygen index calculated as follows: ((FiO2) x (Mean airway pressure)) / (PaO2)	**2016: Unknown Status, last update January 2016 (information assessed on clinical.trials.gov).**<br><br>- Secondary outcomes days 3, 14, and 28 after MSC: a) <u>Lung mechanics</u>, including arterial oxygen saturation, tidal volume, minute ventilation, and ratio PaO2/FiO2, b) <u>hemodynamic parameters</u>, including systolic, diastolic, and mean arterial blood pressure amount of required vasopressor heart rate, c) <u>Plasma cytokines</u>, including IL-1, IL-6, IL-8, IL-10, d) <u>Markers of inflammation and infection</u>, including lactate, DIC score, SOFA score, C-reactive protein, & procalcitonin, e) <u>Ventilator weaning parameters,</u> including failure of ventilator weaning, weaning time, and ventilation time, and<br><br>f) <u>Mortality at day 14 and 28</u>, defined as death by any cause, and g) <u>ICU and hospital stay at day 28</u>, expressed as total duration of ICU or hospital stay.
**Allogeneic UC-MSCs**<br><br>(Umbilical cord (UC)-derived mesenchymal stromal cells)	**NCT02444455**<br><br>**Indication ALI and ARDS**<br><br>(UCMSC-ALI in collaboration with the Ivy Institute of Stem Cells Co. Ltd)	Affiliated Hospital to Academy of Millitary Medical Sciences, Bejing, China	Phase I-II, open label, controlled prospective study to assess safety and efficacy of UC-MSCs in patients with ALI/ARDS (phase I ALI and phase II ARDS) in addition to standard of care (SoC).<br><br>**Dosing:** 0.5mio/kg once a day and a total of 3 times.<br><br>**Estimated recruitment of 20 participants between May 2015 and March 2017, with completion in December 2017.**	- Primary outcome measure is safety as determined by assessment of major adverse events (Time frame: day 0 the start of treatment to day 14), defined as death and the incidence of prespecified infusion-associated events and non-serious adverse events thought to be related to MSC infusion.	**2015: Unknown Status, last update May 2015 (information assessed on clinical.trials.gov).**<br><br>- Secondary outcomes:<br><br>a) <u>Quantify pulmonary respiratory function</u>: at day 2, 7, and 14, measured by chest computerized tomography,<br><br>b) <u>Arterial blood gas analysis</u>: at day 2, 7, and 14, to assess efficacy of UC-MSCs,<br><br>c) <u>Biological markers of inflammation:</u> IL-6 and IL8 at 6-hours post MSC-infusion and at day 1, 2, and 3, to assess efficacy of UC-MSCs.

**Allogeneic AT-MSCs**<br><br>(Adipose tissue derived MSCs, Cx611 product, provided by the sponsor TiGenix now Takeda)	**NCT02328612**<br><br>**Indication LPS-Induced human sepsis model** (to assess safety of AT-MSCs given by IV-infusion)		Randomized, single-blind, parallel group, placebo-controlled trial; Simulation of sepsis by IV injection of LPS (2ng/kg) into healthy volunteer subjects 1-hour after Cx611 treatment; **Recruitment of n=32 healthy subjects with four treatment arms:** placebo or allogeneic AT-MSCs IV at either 0.25, 1.0, or 4.0 mio/kg.<br><br>**Study start Oct 2014, completion March 2015.**	- Primary outcome to study the effect of Cx611 on the inflammatory response to intravenous LPS in humans<br><br>- To assess the inflammatory response as measured by laboratory measurements and functional assays of innate immunology (Time frame: change baseline markers up to 10 hours after LPS injection)	**2018: Phase 1 study completed in March 2015 and published online 20th August 2018, accompanied by several mechanistic side-studies (*11-13*).**<br><br>- The infusion of AT-MSCs was well tolerated. The high cell dose of 4mio/kg increased the febrile response and exerted mixed pro-inflammatory (enhanced IL-8 and nucleosome release) and anti-inflammatory effects (increased IL-10 and TGF-beta), and enhanced coagulation activation and reduced fibrinolytic response. Blood leukocyte transcriptome analysis showed a biphasic effect of AT-MSCs on the LPS response. In murine model side studies AT-MSCs reduced bacterial burden, lung inflammation and attenuated lung pathology, but did not influence distant organ injury. FROZEN AT-MSCs increased systemic clotting due to tissue factor (TF/CD142).
**Allogeneic AT-MSCs**<br><br>(Adipose tissue derived MSCs, Cx611 product, provided by the sponsor TiGenix now Takeda)	**NCT03158727**<br><br>**Indication sepsis/bacterial pneumonia**<br><br>(SEPCELL Study)	N=33 European study locations	Randomized, multicenter, double-blind, placebo-controlled phase 1b/II trial to assess the safety, tolerability and efficacy of IV AT-MSCs (Cx611) as adjunctive therapy in addition to standard of care (SoC) therapy, to patients with severe community-acquired bacterial pneumonia;<br><br>**Planed recruitment of n=180 participants;**<br><br>**Study start 30th of January 2017, estimated completion April 2022.**	- Primary objective to study safety profile of two allogeneic Cx611 infusions within 3 days (on day 1 and 3) at a dose of 160mio cells each to monitor any adverse event and immunological host response during 90days follow-up	**2020: Clinical trial recruiting no publications yet (information assessed on clinical.trials.gov).**<br><br>- Secondary study objectives: a) reduction of the duration of mechanical ventilation and/or need for vasopressors and/or improved survival, and/or cure of bacterial sepsis, and other efficacy endpoints, b) Mode-of-action of Cx611 in sepsis / bacterial pneumonia by identifying pro-inflammatory and anti-inflammatory pathways

Table S1 Part 2: Selection of representative newly registered MSC and other cell therapy studies for COVID-19 pneumonia.

**Allogeneic BM-MSCs**<br>(Bone marrow (BM)-derived mesenchymal stromal cells)	**ChiCTR2000029990**<br>Ethics committee of the hospital (LL-2020-013-K) issued in Chinese Clinical Trial Registry	China	Pilot trial<br>N=10 confirmed COVID-19 patients<br>7 Intravenous MSCs transplant compared with 3 placebo control	- Safety data - infusional and allergic reactions, secondary infection and life-threatening adverse events<br>- Efficacy data - the level of the cytokines variation, the level of CRP in plasma and the oxygen saturation	**Results** - Pulmonary function and symptoms of 7 patients were significantly improved in 2 days after MSC transplantation. The peripheral lymphocytes were increased, the C-reactive protein decreased, and the over-activated cytokine-secreting immune cells CXCR3+CD4+ T cells, CXCR3+CD8+ T cells, and CXCR3+ NK cells disappeared in 3-6 days, while a group of CD14+CD11c+CD11bmid regulatory DCs dramatically increased. The level of TNF-α was decreased, while IL-10 increased in MSC treatment group compared to the placebo control group.
**Allogeneic BM-MSCs**<br>(Bone marrow derived mesenchymal stromal cells)	**ChiCTR2000029990**<br>https://apps.who.int/trialsearch/Trial2.aspx?TrialID=ChiCTR2000029990	China	Phase 1+ 2 study<br>N=120 confirmed COVID-19<br>MSCs - 60; control group – 60	Improved respiratory system function (blood oxygen saturation) recovery time;	**Recruiting**<br>Date of first enrolment: 2020-01-30
**Allogeneic UC-MSCs**<br>(Umbilical cord (UC)-derived mesenchymal stromal Cells)	**NCT04269525**	Wuhan, China	Phase 2 study<br>N=10 pts. serious or critical COVID-19.<br>Intravenous UC-MSCs on day 1, 3, 5, and 7 after enrollment, once per day.	Oxygenation index (Time Frame day 14)<br>partial arterial oxygen pressure (PaO2) / oxygen concentration (FiO2)	**Recruiting**<br>Estimated Primary Completion Date / Estimated Study Completion Date:<br>April 30, 2020 / September 30, 2020
**Unspecified mesenchymal stromal cells**	**NCT04252118**	China	Phase 1 open-label, non-randomized, intervention<br>N=20 pts. with COVID-19:<br>N=10 MSCs vs N=10 conventional standard of care treatment (control)	- Size of lesion area by chest radiograph or CT (Time Frame day 28)<br>- Side effects in the MSCs treatment group (Time Frame day 180)	**Recruiting**<br>Estimated Primary Completion Date / Estimated Study Completion Date:<br>December 2020 / December 2020

**Allogeneic UC-MSCs**<br>(Umbilical cord (UC)-derived mesenchymal stromal Cells)	**NCT04273646**	Wuhan, China	Open label, randomized, Intervention study<br>N=48 pts. with severe COVID-19, randomized to MSCs Treatment or placebo	- Pneumonia severity index (Time Frame 0 - 12 week)<br>Oxygenation index (PaO2/FiO2) (Time Frame 0 - 12 week)	**Not yet Recruiting**<br>Estimated Primary Completion Date / Estimated Study Completion Date:<br>June 30, 2020 / February 15, 2022
**Allogeneic AT-MSC-EVs**<br>(Aerosol inhalation of exosomes from adipose tissue (AT)-derived mesenchymal stromal cells)	**NCT04276987**	China	Phase 1, open label, pilot study<br>N=30 pts. with severe COVID-19<br>Single group assignment	- Adverse reaction (AE) and severe adverse reaction (SAE) (Time Frame day 28)<br>Time to clinical improvement (TTIC) (Time Frame day 28)	**Not yet Recruiting**<br>Estimated Primary Completion Date / Estimated Study Completion Date:<br>May 31, 2020 / July 31, 2020
**Allogeneic PT-MSCs**<br>(Wharton's Jelly-derived mesenchymal stromal cells)	**NCT04313322**	Jordan	Phase 1, open label, Interventional study<br>N=5 pts. with COVID-19<br>Single group assignment	-Clinical outcome (3 week)<br>- CT scan (3 week)<br>RT-PCR (3 week)	**Recruiting**<br>Estimated Primary Completion Date / Estimated Study Completion Date:<br>June 30, 2020 / September 30, 2020
**Stem Cell Educator (SCE)**	**NCT04299152**	China	Phase 2, randomized, intraventional study<br>N=20 pts. with severe ARDS, randomized to SCE Treatment or placebo	Determine the number of Covid-19 patients who were unable to complete SCE Therapy (4 week)	**Not yet Recruiting**<br>Estimated Primary Completion Date / Estimated Study Completion Date:<br>October 9, 2020 / November 10, 2020

**Unspecified mesenchymal stromal cells**	**NCT04288102**	China	Phase 1 (solution of MSCs) + phase 2(MSCs), randomized, multicenter, intraventional study<br><br>N=60 pts. with severe COVID-19, randomized to MSCs Treatment or placebo	-Improvement time of clinical critical treatment (28 days)<br>- Side effects in the MSCs treatment group (Time Frame day 28)	**Recruiting**<br><br>Estimated Primary Completion Date / Estimated Study Completion Date:<br><br>December 31, 2020 / December 31, 2021
**Allogeneic MSCs**<br><br>(Human menstrual blood-derived stromal cells)	**ChiCTR2000029606**<br><br>http://www.chictr.org.cn/showprojen.aspx?proj=49146	Hangzhou China	Open label, 5 arm study.<br><br>N=30 with severe and critically COVID-19<br><br>Randomized to stem cells, Conventional Control, Artificial liver therapy, Artificial liver therapy + stem cells or Conventional Control	Mortality	**Recruiting**<br><br>From 2020-01-15To 2022-12-31
**UCB-MNCs**<br><br>(Umbilical cord blood (UCB) mononuclear cells)	**ChiCTR2000029569**<br><br>https://apps.who.int/trialsearch/Trial2.aspx?TrialID=ChiCTR2000029569	Hubei, China	Open label<br><br>N=30 with severe and critically COVID-19<br><br>Randomized to stem cells or Conventional Control	PSI	***Pending***<br><br>Date of first enrolment: 2020-02-05
**UCB-MNCs**<br><br>(Umbilical cord blood (UCB) mononuclear cells)	**ChiCTR2000029572**<br><br>http://www.chictr.org.cn/showprojen.aspx?proj=41760	Hubei, China	Open label<br><br>N=30 with severe and critically COVID-19<br><br>Randomized to stem cells or Conventional Control	PSI	**Recruiting**<br><br>From2020-02-05To 2021-04-30

**Cord Blood NK Cells Combined with Cord Blood Mesenchymal Stem Cells**	ChiCTR2000029817<br><br>http://www.chictr.org.cn/historyversionpuben.aspx?regno=ChiCTR2000029817	Guangzhou, China	Open label.<br><br>N=60 with COVID-19<br><br>Randomized to high dose NK cells with stem cells, Conventional dose NK cells and mesenchymal stem, preventive dose NK cells and mesenchymal stem	Time to disease recovery	***Pending***<br><br>From 2020-02-20To 2021-02-20
**NK cells**	**NCT04280224**	Henan, China	Phase 1, open label, randomized, Interventional study<br><br>N=30 pts. with COVID-19<br><br>NK cells Treatment or Conventional Control	- Improvement of clinical symptoms including duration of fever<br>- Improvement of clinical symptoms including respiratory frequency<br>Number of participants with treatment-related adverse events evaluated with CTCAE,version 4.0	**Recruiting**<br><br>Estimated Primary Completion Date / Estimated Study Completion Date:<br><br>September 30, 2020 / December 30, 2020

REFERENCES

1. O. Ringden, Mesenchymal stem (stromal) cells for treatment of acute respiratory distress syndrome. *The lancet. Respiratory medicine* **3**, e12 (2015).
2. O. Ringdén *et al.*, Successful Reversal of Acute Lung Injury using Placenta-Derived Decidual Stromal Cells. *Journal of Stem Cell Research & Therapy* **4**, 244-249 (2014).
3. O. E. Simonson *et al.*, In Vivo Effects of Mesenchymal Stromal Cells in Two Patients With Severe Acute Respiratory Distress Syndrome. *Stem cells translational medicine* **5**, 845 (2016).
4. J. E. Gotts, M. A. Matthay, Treating ARDS: new hope for a tough problem. *The lancet. Respiratory medicine* **2**, 84-85 (2014).
5. K. D. Liu *et al.*, Design and implementation of the START (STem cells for ARDS Treatment) trial, a phase 1/2 trial of human mesenchymal stem/stromal cells for the treatment of moderate-severe acute respiratory distress syndrome. *Ann Intensive Care* **4**, 22 (2014).
6. J. Walter, L. B. Ware, M. A. Matthay, Mesenchymal stem cells: mechanisms of potential therapeutic benefit in ARDS and sepsis. *The lancet. Respiratory medicine* **2**, 1016-1026 (2014).
7. J. Wilson, D. McKenna, K. D. Liu, M. A. Matthay, Mesenchymal stem (stromal) cells for treatment of acute respiratory distress syndrome - authors' reply. *The lancet. Respiratory medicine* **3**, e12-13 (2015).
8. M. A. Matthay *et al.*, Treatment with allogeneic mesenchymal stromal cells for moderate to severe acute respiratory distress syndrome (START study): a randomised phase 2a safety trial. *The lancet. Respiratory medicine* **7**, 154-162 (2019).
9. H. Zhang, Y. Li, A. S. Slutsky, Precision medicine for cell therapy in acute respiratory distress syndrome. *The lancet. Respiratory medicine* **7**, e13 (2019).
10. M. A. Matthay, H. Zhuo, J. E. Gotts, K. D. Liu, C. S. Calfee, Precision medicine for cell therapy in acute respiratory distress syndrome - Authors' reply. *The lancet. Respiratory medicine* **7**, e14 (2019).
11. D. Perlee *et al.*, Intravenous Infusion of Human Adipose Mesenchymal Stem Cells Modifies the Host Response to Lipopolysaccharide in Humans: A Randomized, Single-Blind, Parallel Group, Placebo Controlled Trial. *Stem Cells* **36**, 1778-1788 (2018).
12. D. Perlee *et al.*, Human Adipose-Derived Mesenchymal Stem Cells Modify Lung Immunity and Improve Antibacterial Defense in Pneumosepsis Caused by Klebsiella pneumoniae. *Stem cells translational medicine* **8**, 785-796 (2019).
13. D. Perlee *et al.*, Role of tissue factor in the procoagulant and antibacterial effects of human adipose-derived mesenchymal stem cells during pneumosepsis in mice. *Stem cell research & therapy* **10**, 286 (2019).
